# Anaesthetic Management of Hermansky-Pudlak Syndrome with Major Hemorrhage: Based on a Case Report

**DOI:** 10.4274/TJAR.2026.252131

**Published:** 2026-06-26

**Authors:** Akın Akbulut, Doruk Yaylak, Yasemin Sincer, Yavuz Gürkan

**Affiliations:** 1 Koç University Faculty of Medicine Department of Anaesthesiology and Reanimation, İstanbul, Türkiye

**Keywords:** Hermansky-Pudlak syndrome, hemodynamics, paediatric anaesthesia, blood coagulation disorder, orthopaedic anaesthesia

## Abstract

Hermansky-Pudlak Syndrome (HPS) is a rare autosomal recessive disorder characterized by oculocutaneous albinism, platelet dysfunction, and bleeding diathesis. This case discusses the anaesthetic and hemostatic management of a  paediatric patient with HPS undergoing scoliosis surgery, which has not been previously documented. A 15-year-old female patient with HPS presented with scoliosis requiring surgical correction. Key clinical findings included albinism, neutropenia, cardiovascular abnormalities, and prolonged platelet function test results. Preoperative assessments identified significant risks of bleeding and pulmonary complications, necessitating multidisciplinary planning. Intraoperative interventions included the administration of 1-desamino-8 D-arginine vasopressin, tranexamic acid, and blood product transfusions to manage intraoperative hemorrhage. Postoperatively, the patient required intensive care support and was discharged without further complications. This case emphasizes the importance of early diagnosis, preoperative optimization, and individualized anaesthetic and hemostatic management for patients with HPS undergoing major surgery. Multidisciplinary collaboration and vigilance are essential to mitigating bleeding risks and ensuring favorable outcomes in this high-risk population.

**Main Points**

• Hermansky-Pudlak Syndrome (HPS) presents significant perioperative challenges due to platelet dysfunction and bleeding diathesis, particularly in major surgeries such as scoliosis correction.

• Multidisciplinary preoperative planning, including specialists in hematology, pulmonology, and anaesthesiology, is critical for assessing bleeding risk and pulmonary complications in patients with HPS.

• Intraoperative administration of vasopressin, tranexamic acid, and targeted blood-product transfusions can effectively manage hemorrhagic complications in HPS.

• Early diagnosis, individualized anaesthetic strategies, and vigilant perioperative care are essential for successful surgical outcomes in paediatric patients with HPS.

## Introduction

Hermansky-Pudlak Syndrome (HPS) is a rare autosomal recessive disease with a prevalence of 1-9 per 1,000,000 individuals worldwide.^1^ The disease’s clinical manifestations primarily arise from defects in the formation and function of lysosome-related organelles, including melanosomes and platelet dense granules, due to a shared protein deficiency. It is characterized by oculocutaneous albinism; nystagmus; bleeding diathesis; platelet dysfunction; progressive pulmonary fibrosis*; *granulomatous colitis; immunodeficiency; and renal and cardiovascular pathologies.^2^ Bleeding and respiratory problems constitute the main challenges in anaesthesia management.^3^

HPS often presents in childhood with symptoms such as easy bruising, nosebleeds, or prolonged bleeding after surgery. Despite having normal platelet counts, patients experience extended bleeding times.^2^ Anaesthetic management and surgical procedures for patients with HPS require special considerations.^4^ Preoperative optimization, including considerations for platelet transfusions or prophylactic administration of 1-desamino-8 D-arginine vasopressin (DDAVP), is recommended to enhance patient outcomes and mitigate potential perioperative complications.^5^

In this case, we aimed to present the anaesthetic management of bleeding in a  paediatric patient with HPS who underwent scoliosis surgery in our institution in November 2023. Although some anaesthetic management strategies are specific to gynecological cases, to the best of our knowledge, this is the first reported case in the literature addressing the management of bleeding caused by a musculoskeletal disorder in a patient with HPS.^6, 7, 8, 9^

## Case Report

A 15-year-old girl diagnosed with a genetic defect in the *AP-3* gene of HPS type 2 (HPS-2), weighing 22.25 kg and measuring 134 cm in height, both below the second percentile, was scheduled for scoliosis surgery. The patient had a history of safe anaesthesia, including patent ductus arteriosus (PDA) ligation and aortic coarctation repair at 3.5 months of age, followed by residual PDA closure via transcatheter intervention at 2 years of age.

**Figure 1:**
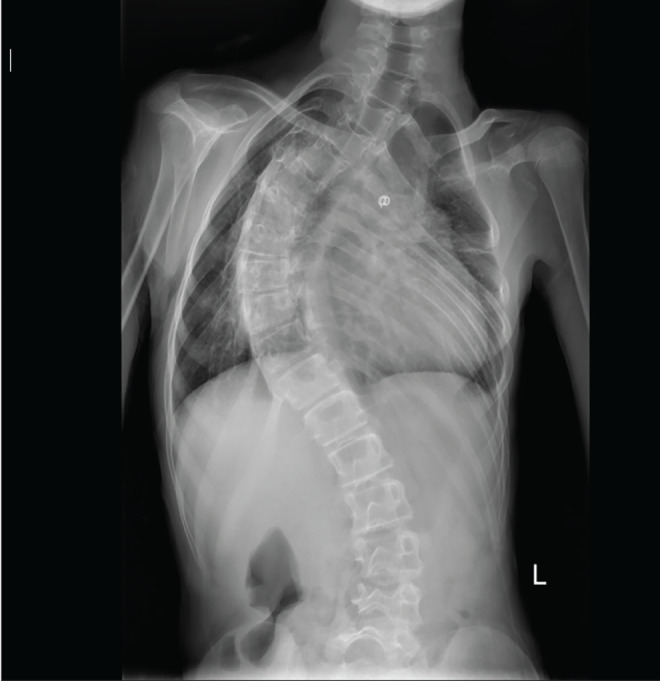
Chest X-ray revealing advanced scoliosis of the patient.

The patient did not have a prominent bleeding event in her medical history and was currently receiving filgrastim 300 µg three times a week. The preoperative complete blood count revealed neutropenia, while coagulation parameters were within the normal range (neutrophil: 0.5 K mcL^-1^, hemoglobin: 14 g dL^-1^, platelet: 182 K mcL^-1^). The chest X-ray revealed advanced scoliosis (Figure 1). Paediatric pulmonology and hematology consultations were conducted preoperatively. Thrombocyte function tests (platelet function analyzer-200) revealed prolonged bleeding times for collagen/epinephrine (>300 seconds; normal: 85-157 seconds) and collagen/adenosine diphosphate (ADP) (253 seconds; normal: 65-125 seconds). Hematology advised that reserved platelet concentrates be prepared due to the risk of prolonged bleeding during and after surgery, even in the absence of dental, gingival, or mucosal bleeding. The pulmonology service recommended pre- and postoperative blood gas monitoring for  paediatric patients who are unable to perform pulmonary function tests; noninvasive ventilation if needed; and  paediatric intensive care unit (PICU) respiratory support if required.

**Figure 2:**
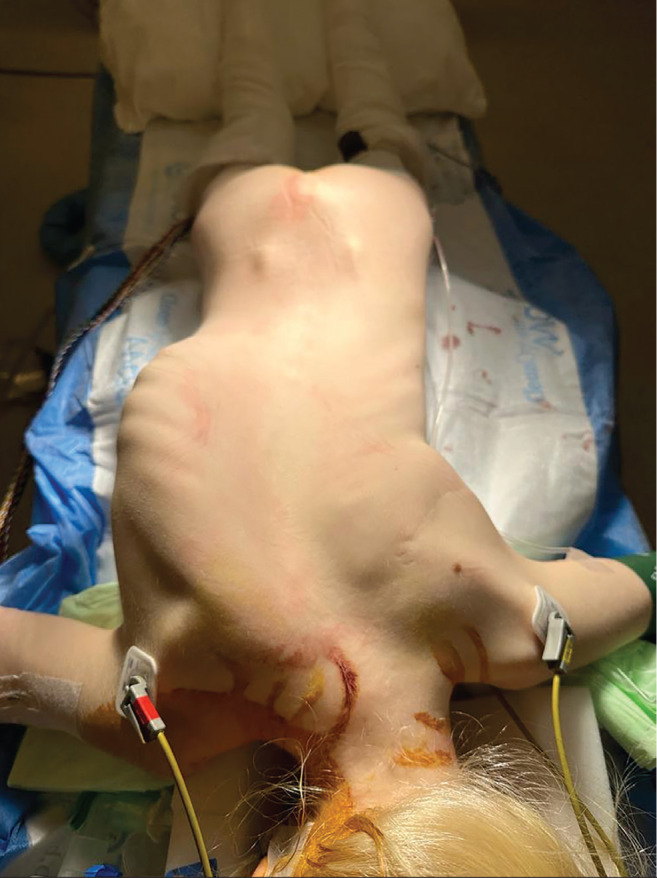
The position of the patient during surgery.

After obtaining the informed consent for publication from the patient’s parents, the patient was transferred to the operating room. Premedication was administered as an intravenous (IV) dose of 1 mg of midazolam, followed by induction of anaesthesia with propofol (2 mg kg^-1^), fentanyl (1 µg kg^-1^), and rocuronium (0.5 mg kg^-1^). Intubation was successfully performed with a 5.0 cuffed endotracheal tube without complications. A central jugular venous catheter and a right radial arterial catheter were inserted after induction. General anaesthesia was maintained with continuous IV infusion of propofol (0.03-0.06 mg kg^-1^ h^-1^) and remifentanil (0.03-0.06 µg kg^-1^ h^-1^). The patient was turned to the prone position (Figure 2). After the first incision at the T2-L3 level, a tendency to bleed was observed.

IV fluid replacement was initiated via the central venous line, and DDAVP 6 µg  IV and tranexamic acid 20 mg kg^-1^ IV bolus were administered immediately. A tranexamic acid infusion at 10 mg h^-1^ IV was started. Due to low blood pressure, a noradrenaline IV infusion at a rate of 0.3 µg kg^-1 ^h^-1^ was initiated. Three units of erythrocyte suspension, one unit of fresh frozen plasma, and one unit of pooled platelet concentrate were used throughout the procedure, with 2500 mL of IV crystalloid administered in total. The procedure lasted five hours, during which a total blood loss of 3000 mL was detected.

The patient was transferred to the PICU postoperatively, where she was intubated and sedated for three days. Her hemodynamics remained stable, and the noradrenaline infusion was discontinued on postoperative day two. The patient was successfully discharged from the PICU to the surgical ward without further complications. No bleeding issues were recorded during her stay in the hospital ward. Postoperative transfusions included three units of erythrocyte suspension, three units of fresh frozen plasma, and two units of platelet concentrate. Additional replacements included cryoprecipitate and vitamin K. No further episodes of thrombocytopenia were observed.

## Discussion

HPS, particularly the AP-3 (HPS2) subtype (AP3B1), is associated with cellular abnormalities, including defects in melanosomes, dense granules, and Weibel-Palade bodies, leading to manifestations such as pulmonary fibrosis, oculocutaneous albinism, bleeding diathesis, and immunodeficiency. Additional complications include interstitial lung disease, periodontitis, and increased susceptibility to infections.^10^ Platelet dense granules contain serotonin, ADP, and calcium, and their deficiency results in prolonged bleeding times and abnormal platelet aggregation tests. Electron microscopy is particularly useful in diagnosing dense granule deficiencies.^2^

Mucocutaneous bleeding is common in HPS, and DDAVP shortens bleeding time by increasing plasma levels of Factor VIII and von Willebrand factor. Antifibrinolytics, such as tranexamic acid, are also beneficial. Preoperative assessment and perioperative platelet transfusion are critical to mitigating bleeding risk. Despite variable efficacy, DDAVP and platelet transfusions are often effective in surgical contexts.^2^

The anaesthetic management of HPS requires special considerations, as bleeding times may be prolonged even with normal platelet counts. Preoperative hematology consultation is essential for optimizing outcomes.^7^ Prophylactic DDAVP and platelet transfusion significantly reduce perioperative blood loss, although responses to DDAVP vary.^4, 5^ Recombinant factor VIIa may be an option for patients who are unresponsive to DDAVP.^5^

Studies show that platelet transfusions effectively limit blood loss, whereas outcomes with DDAVP are less consistent.^6, 11^ Vigilant perioperative planning is crucial, as untreated bleeding can lead to severe complications.^12^ This case underscores the importance of early diagnosis, individualized hemostatic management, and multidisciplinary collaboration. Pre-procedural pulmonary assessments also help address ventilation challenges and ensure stable postoperative outcomes.

Managing HPS during surgery demands meticulous planning and tailored therapeutic strategies. While platelet transfusions are the cornerstone of hemostatic management, variability in DDAVP efficacy highlights the need for individualized care.

## Ethics

**Informed Consent:** Informed consent for publication was obtained from the patient’s parents.
